# Synergistic Role of Oxidative Stress and Blood-Brain Barrier Permeability as Injury Mechanisms in the Acute Pathophysiology of Blast-induced Neurotrauma

**DOI:** 10.1038/s41598-019-44147-w

**Published:** 2019-05-22

**Authors:** Matthew Kuriakose, Daniel Younger, Arun Reddy Ravula, Eren Alay, Kakulavarapu V. Rama Rao, Namas Chandra

**Affiliations:** Center for Injury Biomechanics, Materials and Medicine (CIBM3), Department of Biomedical Engineering, New Jersey Institute of Technology, Newark, NJ 07102-1982 USA

**Keywords:** Blood-brain barrier, Neuroscience, Blood-brain barrier, Blood-brain barrier, Neuroscience

## Abstract

Blast-induced traumatic brain injury (bTBI) has been recognized as the common mode of neurotrauma amongst military and civilian personnel due to an increased insurgent activity domestically and abroad. Previous studies from our laboratory have identified enhanced blood-brain barrier (BBB) permeability as a significant, sub-acute (four hours post-blast) pathological change in bTBI. We also found that NADPH oxidase (NOX)-mediated oxidative stress occurs at the same time post-blast when the BBB permeability changes. We therefore hypothesized that oxidative stress is a major causative factor in the BBB breakdown in the sub-acute stages. This work therefore examined the role of NOX1 and its downstream effects on BBB permeability in the frontal cortex (a region previously shown to be the most vulnerable) immediately and four hours post-blast exposure. Rats were injured by primary blast waves in a compressed gas-driven shock tube at 180 kPa and the BBB integrity was assessed by extravasation of Evans blue and changes in tight junction proteins (TJPs) as well as translocation of macromolecules from blood to brain and vice versa. NOX1 abundance was also assessed in neurovascular endothelial cells. Blast injury resulted in increased extravasation and reduced levels of TJPs in tissues consistent with our previous observations. NOX1 levels were significantly increased in endothelial cells followed by increased superoxide production within 4 hours of blast. Blast injury also increased the levels/activation of matrix metalloproteinase 3 and 9. To test the role of oxidative stress, rats were administered apocynin, which is known to inhibit the assembly of NOX subunits and arrests its function. We found apocynin completely inhibited dye extravasation as well as restored TJP levels to that of controls and reduced matrix metalloproteinase activation in the sub-acute stages following blast. Together these data strongly suggest that NOX-mediated oxidative stress contributes to enhanced BBB permeability in bTBI through a pathway involving increased matrix metalloproteinase activation.

## Introduction

Blast-induced traumatic brain injuries (bTBIs) are the most prevalent form of neurotrauma amongst soldiers and some in civilian populations due to asymmetric warfare with increased acts of terrorism domestically and abroad^1–6^. In spite of the increase in blast-induced neurotrauma (BINT) studies, there are still significant gaps in the understanding of how the acute shockwave induced biomechanical injury plays a role in the subsequent secondary biochemical injuries in the chronic stages. A recent survey reported that more than 30 phase III clinical trials aimed at targeting TBI have failed and, to this effect, our group has attempted to elucidate the temporal and spatial neuropathology of bTBI, in relation to blast-overpressure, as it pertains to two commonly identified pathological factors: oxidative stress increase and blood-brain barrier (BBB) breakdown^7–10^.

Oxidative stress has been implicated in multiple modes of TBI^11,12^ and is mainly induced by reactive oxidative species (ROS). These include, but are not limited to, superoxide (O2⋅−), hydroxyl radical (HO⋅), and hydrogen peroxide (H_2_O_2_)^13,14^. While a basal level of ROS is to be expected to occur during normal reactions in redox-reactions during and the electron transport chain operation, an excess amount, as seen after injury, can be harmful. NADPH oxidase (NOX) is a multi-subunit enzyme that catalyzes the formation of superoxide radicals from available molecular oxygen. As previously reported by our group and other investigators^15–17^, NOX is upregulated in multiple brain regions following blast injury and neurons maximally contribute to the highest increase in hippocampus compared to other neural cells. It is also worth noting that a single blast substantially increases superoxide levels in multiple brain regions from the pre-frontal cortex to hippocampus to cerebellum, all along the direction of shock wave propagation^17^.

We also recently identified that direct blast biomechanical loading causes disruption of brain vascular integrity (BBB) in multiple brain regions within minutes of loading and that this BBB disruption further increased in four hours following blast before returning to normal at 24 hours post-injury^18^. The BBB is a selectively-permeable membrane separating the brain from the circulatory system, consisting of tight junction complexes, which attach adjacent endothelial cells together, as well as a host of modulating neural cells including pericytes and astrocytes, which wrap around the endothelium. In a recent work by our group, increased permeability of the BBB was most significantly seen four hours post-blast across the cerebral hemisphere, with highest permeability seen in the frontal cortex^18^. Damage to the BBB was established through extravasation of tracers Evans blue and sodium fluorescein, the dislodging of tight junction proteins, and leakage of blood-borne cells into the brain parenchyma and brain parenchymal molecules into the blood^18^. Increased permeability of the BBB has been observed in several other animal models of TBI including closed cortical injuries^19–21^, weight drop models^22,23^, as well as blast models^24–29^.

Oxidative stress is a well-known factor for BBB disruption in blunt TBI as well as other neurological disorders including Alzheimer’s and Parkinson’s diseases^30–32^. However, no studies have been performed on the interaction between oxidative stress and BBB permeability changes in blast TBI. Since our recent studies indicate blast injury results in disruption of BBB and activation of factors conducive to oxidative stress^17,18^, we sought to examine the interaction of these two well-known pathogenic factors in BINT. We hypothesize that while BBB is acutely disrupted immediately following blast injury through biomechanical means, and enhanced BBB disruption in later stages (zero to four hours) is due, at least in part, to the activation of NADPH oxidase (NOX) and associated increase in oxidative stress. This study therefore employed apocynin, which is known to block the assembly of NOX subunits and prevent activation of NOX, and reduce superoxide production to ascertain if NOX plays a role in the manifestation of BBB breakdown. Apocynin was used to examine the role of NOX on BBB disruption and not as a therapeutic agent though such a treatment possibility exists; however, that is not the purpose of the present study.

## Materials and Methods

### Animal preparation

Adult (10-week old), male Sprague-Dawley rats (Charles River Laboratories) weighing between 350 ± 50 g were used in accordance with protocols approved by Rutgers University Institutional Animal Care and Use Committee (IACUC). Animals were housed at 22 °C with free access to food and water in a 12 hour light-dark cycle. Animals were divided among sham, blast, and treatment groups, all to be sacrificed 15 min or four hours post-blast (or, in the case of sham animals, with only noise exposure). All methods used throughout the study were performed in accordance with protocols, guidelines, and regulations approved by Rutgers University IACUC.

### Exposure to blast and apocynin pre-treatment

Rats were exposed to a single shock wave at the New Jersey Institute of Technology (Center for Injury Biomechanics, Materials, and Medicine) in the shock tube described in previous publications^16,33–35^. A total of 54 rats were used in the study for different experimental methods with at least 4–5 animals in each experimental group (power value of 0.8, α = 0.05) based on a priori power analysis. Prior to blast exposure, all animals (controls, blast, blast + apocynin) were anesthetized with 5% isoflurane, released in a chamber containing 95% air and 5% CO_2_, until rats were unresponsive to noxious stimulation. Apocynin (5 mg/kg, Sigma-Aldrich) was administered intraperitoneally 30 min prior to blast exposure. Sham animals were kept near the shock tube to expose them to noise but not any shock wave. A separate group of animals with sham + apocynin treatment was not included in the present study, since several investigators have reported apocynin (5 mg/kg wt) administration to control/sham animals did not exert any toxicity nor affected physiological and behavioral parameters assessed in parallel to animals subjected to traumatic brain injury^36^, splanchnic artery occlusion^37^, liver injury^38^ and experimental epilepsy^39^. Following blast injury, animals were closely monitored for any signs of apnea, loss of motor coordination and neurological severity score (NSS) was evaluated five minutes post exposure. None of the animals included in this study displayed NSS scores that differed from sham animals. Animals were exposed to a single blast of 180 kPa peak overpressure. Prior to transcardial perfusion, animals were anesthetized with a mixture of ketamine and xylazine (1:10 ratio), perfused either 15 min or four hours post-blast. For immunofluorescence and extravasation studies, animals were first perfused with PBS followed by 4% paraformaldehyde, whereas for ELISA studies, animals were perfused with only PBS and brain tissue was collected for preparation of homogenates. Prior to initiation of perfusion, CSF was extracted from the cisterna magna using a 25 gauge butterfly needle from scalp vein set (Exelint International, CA) and was freely allowed to collect into catheter. Blood (about 4 ml) was collected by cardiac puncture (left ventricle) and allowed to settle in vacutainer tubes (BD Bioscience) containing 3.2% sodium citrate for 10 minutes. Plasma was separated from blood by centrifuging at 2000 g. Extravasation of Evans blue was assessed by method recently published by us^18^. All the blast exposures were continuously monitored with high-speed video recording (5000 fps) as described previously^18^ as a quality control measure to ensure that potential secondary/tertiary injuries are absent.

### Immunofluorescence and microscopy

In order to evaluate the increase in NOX1 expression in vascular endothelial cells following blast injury, double immunofluorescence studies were conducted for NOX1 and RECA-1 (a marker of endothelial cells) in the frontal cortex. Briefly, four hours post-injury, both sham and injured animals were transcardially perfused with PBS and brains were fixed with 4% paraformaldehyde (PFA). Tissue section and staining procedure was done in similar manner described in our previous publication^17^. Fixed tissues were incubated overnight at 4 °C with respective primary antibodies to NOX1 (Rabbit polyclonal, Sigma-Aldrich, 1:400) and RECA-1 (mouse monoclonal, Abcam, 1:50). Double immunofluorescence was performed using Alexa Fluor 488 for NOX1 and Alexa Fluor 594 for RECA-1. Quantification of the fluorescence intensities was performed following the method recently published by us^17,18^. Briefly, slides containing mounted coronal sections of frontal cortex were imaged at 20x magnification using a Leica Aperio Versa 200 digital pathology scanner. Control sections were used as reference for adjusting the exposure times and gray scale balance for optimal image quality; once set, these parameters were fixed and used for image acquisition of the remainder of both control and experimental groups. To remove variability that might arise, all sections to be compared were scanned on the same day using the same established scanning protocol. For the immunostained sections, three channels were collected for each coronal section. Blue: 405 nm (DAPI), red: 594 nm (NOX1), and green: 488 nm (RECA-1). For the extravasation studies, two channels were collected. Red: 594 nm (Evans blue) and green: 488 nm (NaFl). Values were expressed as average fluorescence intensity/unit stained area. For the EB extravasation and dihydroethidium (DHE) study we expressed results as percentage change from control due to an absence of a negative control that can be used to remove autofluorescence from the analysis. A maximum intensity threshold was also set to remove any oversaturation from excess fluorescent dye. The algorithm outputs the area of positive staining for each brain region, the average intensity of each channel, and intensity profile of each antigen.

### Superoxide production

Superoxide (O_2._^−^) levels in different brain regions were measured using DHE following the method of Kim *et al*.^39^, and was previously described^17^. Briefly, animals were injected with 5 mg/kg DHE (Molecular probes, MA, dissolved in DMSO) intraperitoneally 30 minutes prior to blast and 4 hours after exposure; animals were transcardially perfused first with PBS followed by 4% PFA (n = 6, per group). Brains were extracted and 50 µm sections were cut using Leica VT 1000S vibratome and mounted. DHE immunofluorescence in each region was visualized and quantified using similar protocol as described above.

### ELISA

As a means to determine the abundance of tight junction proteins and matrix metalloproteinases in the brain tissue, GFAP in the blood plasma, and albumin in the cerebrospinal fluid, ELISAs were performed in the lysates obtained from cerebral hemispheres. Following perfusion with PBS, brains were excised from the skull and cerebrum was homogenized in CellLytic-M (Sigma-Aldrich) using sonicator (Fisher Scientific, IL). Samples were then centrifuged at 14,000 g at 4 °C. The protein concentration in the samples was estimated by bicinochoninic acid (BCA) method (Thermo Scientific, Rockford, IL). Subsequently, samples were diluted in PBS and loaded onto specific ELISA plates for MMP3, MMP9, occludin, and claudin-5 and GFAP (LSBio, Seattle, WA). Plates were read in microplate reader (Spectra Max i3, Molecular Devices) at wavelength of 450 nm. Steps of ELISA were conducted in accordance with manufacturer instructions and samples plotted against a standard curve made up of eight samples using SoftMax Pro 6.5 software.

### *Ex-Vivo* Imaging and Analysis

Slides containing EB extravasated tissue sections were digitized (10x magnification) using Leica Aperio Versa 200 digital pathology grade slide scanner. Fluroescent intensities were quantified after excitation at 594 nm, 125 ms exposure, using AreaQuant, software specifically designed for this imaging application (Leica Biosystems) using similar protocol described in the previous section.

### Statistical analysis

Data are presented as mean ± standard error of the mean. Statistical significance was determined using one-way analysis of variance (ANOVA) to compare mean fluorescence intensities between control, blast, and blast + treatment groups with a post-hoc analysis using Tukey pairwise test to determine differences between individual groups. Normalcy and population variance homogeneity were assessed with Shapiro-Wilk and Levene’s tests respectively. Differences between means were assessed and probability levels of p < 0.05 were considered statistically significant. Minitab 17 Statistical Software was used for all analyses and Origin 2017 was used for generation of bar plots. Bar plots presented in semi-log scale are done so as to capture the intensities when the differences between groups are several orders of magnitude. Fluorescent images were taken using Aperio Versa software and analysis and export done via ImageScope software (LEICA Corp.).

## Results

### NOX1 is upregulated in neurovascular endothelial cells four hours following moderate blast injury

**Figure 1:**
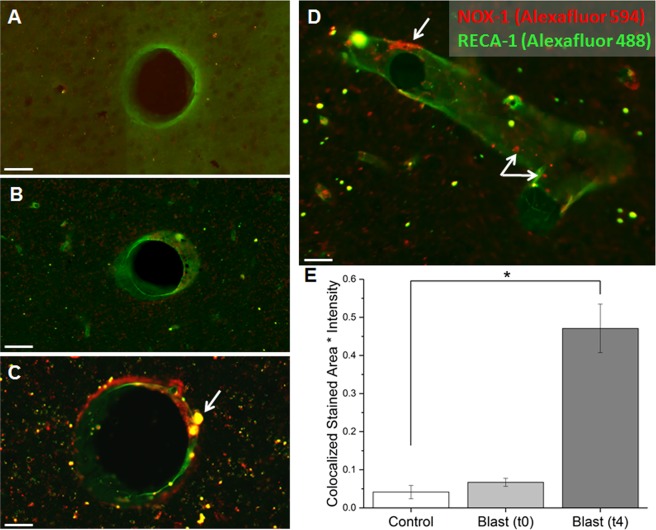
Co-localization of NOX-1 (red) and RECA-1 (green) on vascular endothelial cells in the frontal cortex. (**A**) Controls show negligible NOX-1 on vascular endothelial cells. (**B**) Blast injury after 15 min shows a slight increase in colocalization. (**C**) Four hours following blast, there is a significant upregulation of NOX-1 on marked endothelial cells, with the yellow indicating an overlap of NOX-1 and RECA-1. (**D**) Image showing an alternative view of the vessel in order to show highlight that NOX-1 is upregulated across the length of the vessel lumen, as indicated by the arrows. (**E**) Quantitation of the co-localization between control (n = 5), 15 minutes post-blast n = 5), and 4 hours post-blast groups (n = 5). Scale bars = 30 μm. *Indicates a difference in intensity compared to control with a statistical significance of p < 0.05.

Previous studies in our laboratory identified increased levels of NOX1 and NOX2 in neurons, astrocytes, and microglia following mild blast injury (180 kPa) across the cerebral hemisphere and cerebellum^17^. In the present study, we examined the levels of NOX1 in the vascular endothelial cells in the frontal cortex. The double immunofluorescence for NOX1 and RECA-1 (endothelial cell marker) showed a significant increase in amount of co-localization following blast (Fig. 1). Fifteen minutes post-exposure, there was no change in the fluorescent intensity from controls (Fig. 1B), whereas there was a robust increase (ten-fold) in NOX1 concentration in vascular endothelial cells (Tukey test, p = 0.023) 4 h post-injury. Noteworthy that such increase in NOX1 at 4 h post injury correlated well with our previous observation of highest increase in BBB permeability following blast injury^18^.

### Apocynin significantly reduces superoxide production following blast injury

**Figure 2:**
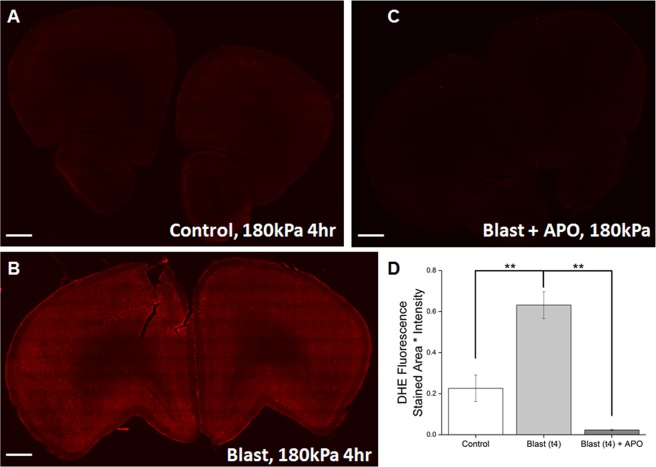
DHE fluorescence in frontal cortex indicating superoxide production. (**A**) Low level of flurescence intensity in control tissue, basal level of superoxide. (**B**) Levels of superoxide robustly increase four hours following blast exposure, (**C**,**D**). Supreoxide is brought back down to basal levels with pretreatment of apocynin Quantitation of DHE fluorescence intensity for the three aforementioned groups. Scale bar = 1 mm. **Indicates a difference between control ((n = 4) and blast groups (n = 4) as well as blast + apocynin group (n = 4) with a statistical significance of p < 0.01.

Several groups have demonstrated that activation of NOX results in increased superoxide production^40,41^. After demonstrating the increase of NOX1 concentration in neurovasculature in the frontal cortex, we sought to determine if this increase leads to an increase of superoxide production. *In-vivo* levels of superoxide were measured using DHE and we observed a clear increase in superoxide produced in the frontal cortex, which not only correlated well with our earlier observation^17^ but is also consistent with the upregulation of NOX1 in the present study (Fig. 2A,B). Differences between control and blast groups (post-ANOVA Tukey test, p = 0.004) and blast and treatment groups (Fig. 2A–C) (Tukey test, p = 0.001) were found to be statistically significant, as seen in Fig. 2D.

### Apocynin significantly attenuates the reduction of tight junction proteins following blast injury

**Figure 3:**
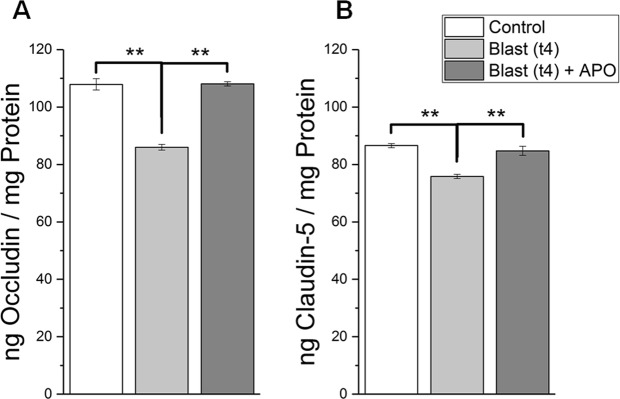
Levels of tight junction proteins, occludin and claudin-5 in control and blast groups as determined by ELISA. (**A**) Shows levels of occludin determined four hours after exposure and compared against controls. (**B**) Shows levels of clasudin-5 in control (n = 5), blast (n = 5), and apocynin treatment groups (n = 5). *Indicates a difference in intensity with a statistical significance of p < 0.05, ** Indicates p < 0.01.

In order to determine the degree of BBB breakdown following blast injury, ELISA was conducted for tight junction proteins occludin and claudin-5 for control, blast, and blast + apocynin groups (Fig. 3). Occludin decreased by 20.3% four hours post-blast compared to controls (ANOVA followed by Tukey test, p = 0.002), but barely changed in animals treated with apocynin (Tukey test, p = 0.946). The difference between the blast group and the treatment group was also significant (Tukey test, p = 0.001). Claudin-5 also displayed a similar trend of decrease in animals four hours post-blast compared to controls (Tukey test, p = 0.001). But similar to occludin, apocynin treatment significantly reversed the reduction of clausin-5 (Fig. 3) as well.

### Apocynin significantly reverses blood-brain barrier permeability changes 4 hours following blast injury

**Figure 4:**
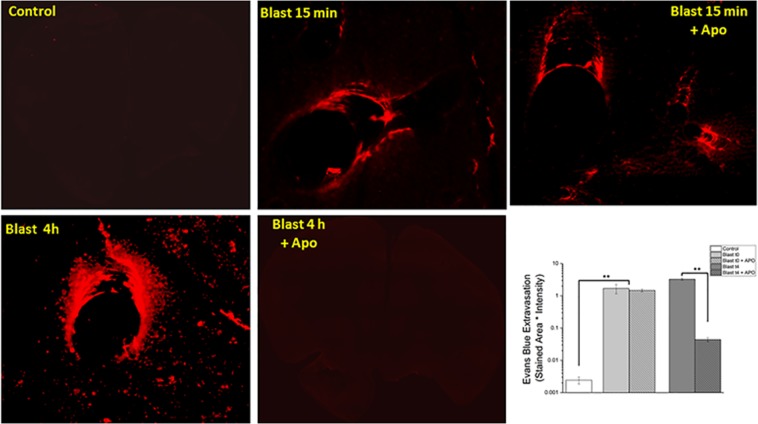
Fluorescence images of Evans blue extravasation. Images at 20x magnification in the frontal cortex in control (n = 4), animals exposed to blast (180 kPa BOP) (n = 4) and apocynin treated groups (n = 4). (**A**) Cortical section of a control animal, with almost no observable extravasation of leakage. Note that blast caused robust extravasation as early as 15 min post-injury and such extravasation was much more pronounced 4 h after the blast injury. A pretreatment with apocynin did not attenuate EB extravasation in animals immediately (15 min) following blast, whereas apocynin completely inhibited the extravasation 4 h post injury, strongly suggesting that acute BBB permeability is a result of direct mechanical loading by stress wave causing vascular rupture. Quantitation of extravasation is shown using a semilog plot in order magnitude differences. Scale bar = 30 μm ** Indicates a difference in intensity with a statistical significance of p < 0.01.

The extent of extravasation was evaluated in the frontal cortex at both fifteen minutes and four hours post-blast injury (Fig. 4). Our group previously displayed that extravasation of Evans blue was significant at both time points at moderate blast injury^18^ and our current results also support our earlier observations. Blast injury caused a significant extravasation in frontal cortex as early as 15 min post injury. Interestingly, apocynin treatment did not attenuate the extravasation at this time point. At four hours post injury, there was a robust extravasation (1300 fold) of EB in the brain parenchyma compared to the control in the frontal cortex (ANOVA followed by Tukey test, p < 0.01) and such extravasation was completely attenuated by treatment with apocynin (p < 0.01).

### Moderate blast induces translocation of glial fibrillary acidic protein into the blood stream

**Figure 5:**
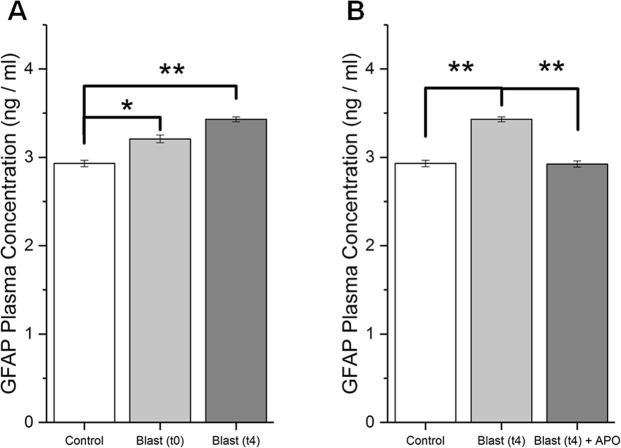
GFAP concentration in blood plasma as determined by ELISA. (**A**) Levels of GFAP in Control (n = 5), 15 minutes post-blast (n = 5) and 4 hours post-blast (n = 5). (**B**) GFAP levels in animals 4 hours following blast with and without apocynin pretreatment (n = 5). * Indicates a difference in intensity with a statistical significance of p < 0.05, ** Indicates p < 0.01.

Concentration of GFAP in the blood plasma was determined by ELISA (Fig. 5). Statistically significant increase in GFAP concentration was seen compared to controls in both blast and apocynin treatment groups 15 minutes after injury (post-ANOVA Tukey test, p = 0.016 and p = 0.002, respectively), whereas apocynin did not alter its levels compared to blast group. At four hours post-blast, there is a further increase in GFAP concentration in blood plasma (Tukey test, p = 0.002), and treatment with apocynin showed a complete restoration of GFAP to that of control levels (Tukey test, p > 0.05). Intergroup comparison reveals a statistically significant difference between blast and treatment groups four hours after injury (Tukey test, p = 0.001).

### CSF-Plasma ratio of albumin increases following moderate blast injury

**Figure 6:**
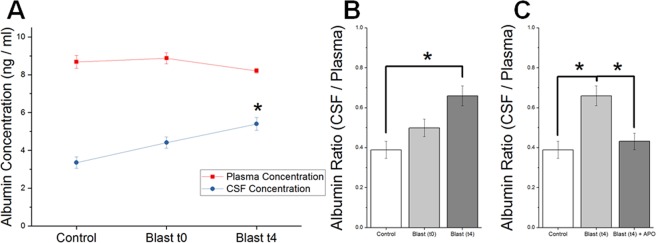
Effect of apocynin on blood-CSF albumin ratio in animals exposed to blast injury. (**A)** Levels of albumin concentration in CSF and blood plasma as determined by ELISA. * Indicates a difference in intensity compared with control with a statistical significance of p < 0.05. (**B,C**) Ratio of albumin concentration in CSF and blood plasma in control (n = 5), 15 min (n = 5) and 4 hours (n = 5) following blast compared with and without apocynin pretreatment (n = 5). * Indicates a difference in intensity with a statistical significance of p < 0.05.

In order to determine the ratio of albumin in the CSF and blood plasma, ELISA was conducted on both samples (Fig. 6). While an increase in the CSF-Plasma albumin ratio was observed acutely (fifteen minutes post-blast) for both blast and treatment groups, statistical significance was not achieved in either (p > 0.05). It should be noted that CSF-plasma ratio is used as a gold standard in clinical settings to determine BBB breakdown^42^. Four hours following blast, a statistically significant increase over control was observed in the injured group (post-ANOVA Tukey test, p = 0.044). Comparison between blast and treatment group at this point also revealed statistically significant difference (Tukey test, p = 0.048).

### Apocynin mitigates the increase of matrix metalloproteinase 3 and 9 following blast injury

**Figure 7:**
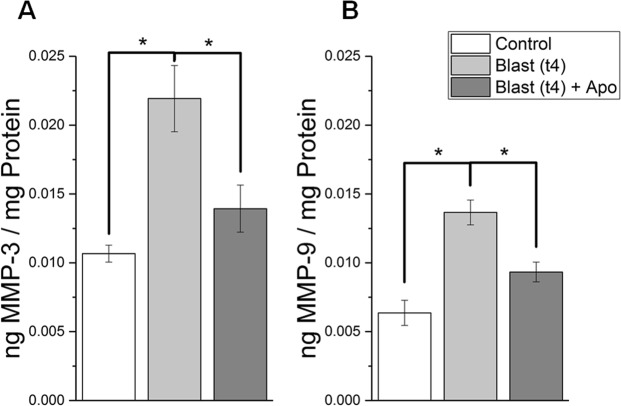
Effect of apocynin on protein levels of matrix metalloproteinases 3 (**A**) and 9 (**B**) in control (n = 5), blast (n = 5), and apocynin treatment groups (n = 5). * Indicates a difference in intensity with a statistical significance of p < 0.05.

In order to examine the role of matrix metalloproteinases in the breakdown of the BBB, we conducted an ELISA for MMPs 3 & 9 for control, blast, and blast + apocynin groups (Fig. 7). MMP3 increased by 105% (ANOVA followed by Tukey test, p = 0.020) compared to controls in the injured group, while the difference between the blast + apocynin and control groups was not statistically significant (Tukey test, p > 0.05). Similarly, MMP9 increased by 115% (Tukey test, p = 0.007) compared to the controls in the injured group, but the difference between blast + apocynin and control groups was not statistically significant (Tukey test, p > 0.05). However, it is important to note that there is still an observed increase in both MMP3 and MMP9 in the blast + apocynin groups, which suggests that NOX activation and subsequent superoxide production *per se* are responsible for MMP activation.

## Discussion

This work aims to investigate the role of NOX-mediated oxidative stress as a mechanism for increased BBB permeability in blast-induced traumatic brain injury, immediately following blast and further increase between blast and 4 hours post-blast. Using apocynin, which inhibits the assembly of various NOX subunits rendering the enzyme inactive, we found that apocynin significantly attenuated the extravasation, preventing the reduction in tight junction proteins as well as reduced the levels of MMPs, between the time points of blast and 4 hours post-exposure. These data together strongly suggest that NOX-mediated oxidative stress significantly contributes to BBB permeability.

**Figure 8:**
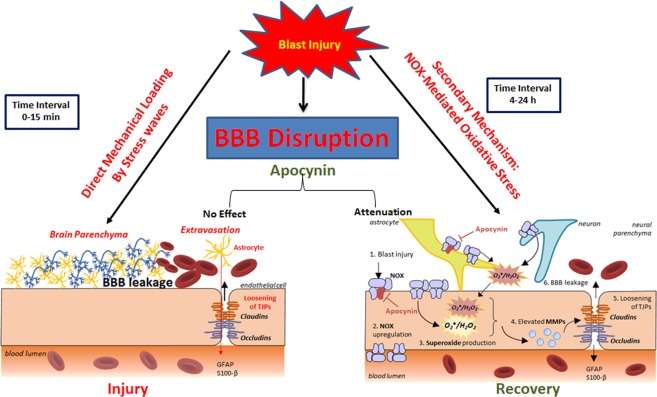
Schematic illustrating the primary and secondary events that contribute to BBB permeability changes following blast TBI. The left panel represents the primary damage caused by direct mechanical loading by stress wave rupturing the vasculature immediately (t0–t15 min) following blast without any biochemical events. The right panel represents secondary biochemical mechanisms such as oxidative stress that are activated by blast, exacerbate the primary mechanical injury to vasculature. Oxidative stress derived by the upregulation of NOX on neurovascular endothelial cells (and other neural cells) cause increased superoxide production both within and outside the endothelial cells. This superoxide elevates MMP production, which breaks down the tight junction complexes connecting adjacent endothelial cells, causing increased permeability of the BBB. Use of apocynin prevents the translocation of the NOX p47 phox subunit, thereby preventing the increase in superoxide production in the later stages of BBB permeability but not immediately after blast.

It should however be noted that in the current study, we employed apocynin as a proof of concept to examine whether oxidative stress contributes to BBB disruption after blast exposure, rather than a therapeutic paradigm. Additionally, we sought to examine whether apocynin has any impact on BBB permeability changes that we earlier observed immediately (15 min) following blast exposure. Accordingly, apocynin was administered 30 min prior to blast exposure. Noteworthy that apocynin did not show any beneficial effects during the acute phase (15 min) of the injury (biomechanical phase) suggesting that oxidative stress and associated effects on BBB permeability are predominantly secondary biochemical factors. As shown in Fig. 8, while the initial disruption of BBB in the acute phase could possibly be attributed to direct mechanical loading on vasculature caused by shockwave, effects seen past the initial stage ought to arise from biochemical effects. As discussed in our earlier work, BBB breakdown can occur both as a direct consequence of mechanical stress wave interaction with vasculature in the immediate stages and continue to increase at 4 h post injury. What we needed to demonstrate is that while apocynin has the ability to reduce oxidative stress induced BBB permeability (secondary) but it could not mitigate the mechanical breakdown. This critical factor would not have been ascertained if the apocynin is administered post-blast.

The pharmacokinetics and the bioavailability of apocynin have been well established. Apocynin appears to be a stable compound once administered into the system with a bioavailability of >8%; half-life of the compound has been determined using LC-MS to be 48 h in the blood circulation^43,44^. A dose of 5 mg/kg employed in the current study also has been used by several investigators to examine the role of NOX-mediated responses in various experimental conditions including stroke^37,45^, and blunt model of TBI^46–51^. A complete restoration of blast-induced BBB permeability at 4-hours’ time point by apocynin strengthens our hypothesis that though initial BBB increase is minimal, sustained BBB disruption is primarily contributed by oxidative stress. However, few studies have reported anti-inflammatory properties of apocynin. Thus, Anter *et al*., ^52^ reported that topical application of apocynin showed anti-inflammatory properties as indicated by reduction in paw swelling in rats. Likewise, another study showed that apocynin significantly reduced the levels of TNF-alpha in animals subjected lateral fluid percussion model of TBI^53,54^. Additionally, diapocynin (a dimer of apocynin) significantly reduced microglial activation in MPTP model of Parkinson disease^55^. Further, apocynin derivative dihydrocoumarin also exerted anti-inflammatory properties *in vitro* in erythrocytes subjected to oxidative injury^56^. Similarly, apocynin has shown anti-inflammatory effects in mouse model of stroke^57^ by reducing the NLRP3 inflammasome. Taken together, these reports also attribute anti-inflammatory actions of apocynin. Noteworthy that we identified robust activation of microglia as early as 4 h after moderate blast injury (180 kPa) (unpublished observation) and that such activation was more pronounced near the site of vascular rupture (increased blood brain barrier permeability) we observed earlier^18^ as well as in the current study. Based on these reports, it is also likely that protective effect of apocynin in the present study may in part mediated by suppressing the neuroinflammation following blast injury.

In the present study, we observed a robust (10-fold) increase in NOX1 expression four hours following blast injury in endothelial cells in the frontal cortex as demonstrated by double immunofluorescence staining of NOX1 with RECA-1, an endothelial cell marker. Our recent observations along with other investigations also report increased activation of NOX1 between four hours and a few days in animal models of TBI^15,17,46,54,58^.

In order to confirm that increased NOX1 expression activates downstream pathways, we examined the superoxide production by prior administration of dihydroethidium (DHE). There was a robust increase in superoxide production in animals subjected to blast injury, which parallels the increased NOX1 expression on vascular endothelial cells. While precise cellular origin of superoxide in the present study is not identified, it is highly likely that the superoxide produced by neural cells including neurons, astrocytes and microglia as a result of increased activation of NOX^17^ may exert a paracrine effect on vascular endothelial cells and cause BBB disruption. Alternatively, vascular endothelial cells *per se* could produce superoxide since our current study display a robust increase in NOX1 levels following blast in these cells, and such superoxide production within the endothelial cells would have caused BBB disruption in an autocrine mechanism. We further established that the superoxide production regardless of its cellular origin, is indeed mediated by NOX1 expression as apocynin inhibits assembly of NOX subunits and renders NOX functionally inactive, completely reducing the production of superoxide consistent with our previous work^17^.

It is interesting to note that our present study observed a close correlation between increased NOX1 expression, its associated increase in superoxide production, and the heightened disruption of the BBB 4 hours after blast injury^18^. The BBB permeability changes were represented not only by the extravasation of EB but also by the reduced levels of tight junction proteins as well as cross translocation of substances between brain and blood and *vice versa*. A significant reduction in both occludin and claudin-5, four hours after blast injury is consistent with extravasation of tracers since loss of tight junction protein integrity is well known to allow leakage of tracers and other blood borne macromolecules into brain parenchyma. Accordingly, increased presence of astrocytic protein, GFAP, in plasma as well as increased CSF-blood ratio of albumin observed in the present study confirm the reciprocal translocation of brain and blood-borne macromolecules as a result of BBB disruption. Noteworthy that a pretreatment with apocynin significantly attenuated the extravasation of tracers, prevented the loss of TJPs and brought back the CSF-blood albumin ratio to that of controls, as well as attenuated increased presence of GFAP in plasma. Together these data again strongly suggest that NOX-mediated oxidative stress contributes to heightened BBB disruption four hours following blast injury.

Several groups have attempted to establish a connection between oxidative stress and BBB breakdown by implicating matrix metalloproteinases^15,59–63^. MMPs are endopeptidases that degrade the extracellular matrix (ECM) in various cell types. While the activity of MMPs are tightly controlled in normal, “uninjured” conditions by endogenous MMP inhibitors, uncontrolled activation has been observed due to oxidative stress^15,64–67^. This in-turn degrades the brain endothelium and substantially increases the permeability of the BBB. The role of MMPs in BBB degradation has been well-investigated^15,59–63^. The present study showed that the levels of both MMP3 and MMP9 significantly increased over controls four hours after blast exposure. Rats pretreated with apocynin displayed significant reduction in the levels of MMPs as compared to animals subjected to blast injury again indicates that NOX-mediated oxidative stress contributes to increase in MMPs, likely leading to degradation of tight junction proteins ultimately causing BBB breakdown.

While it is strongly suggested that oxidative stress is a secondary event that contributes to heightened BBB disruption in the later stages after blast injury, we further extended studies to address whether BBB disruption immediately after blast injury is caused by a direct mechanical loading or in part mediated by any presence of oxidative stress. Accordingly, the ability of apocynin to reduce BBB breakdown only at 4 h post injury but not in the acute phase (prior to 4 h post-injury) in conjunction with our observation of robust increase in NOX1 four hours after blast strongly suggest that oxidative stress appears to be a secondary event occurring following blast injury. Further, our study also strongly suggests that the disruption of BBB immediately after blast is attributable to a direct mechanical loading created by shockwave. Figure 8 illustrates how primary mechanical damage and secondary events such as oxidative stress caused BBB disruption following blast TBI.

It is notable that although we did not observe any changes in the neurological severity scores (NSS) following blast, our recent studies display that either single blast injury at moderate BOP (180 kPa) or repeated (5 successive times) low-level blast (70 kPa) resulted in significant neurobehavioral deficits as identified by anxiety/depression like symptoms, loss of short-term memory as well as sleep disturbances (unpublished observations). Together these observations strongly suggest that (1). Blast induces long-term neurobehavioral deficits and (2). There will be a higher propensity that repeated blast exposures at lower BOP will cumulatively impact on the neurobehavioral changes, in both conditions without any overt NNS NSS change.

In summary, our studies demonstrate that moderate blast injury causes disruption of BBB by loss of TJPs and results in leakage of macromolecules across brain-to-blood as well as increase in NOX expression on vascular endothelial cells and associated increased production of superoxide 4 h after blast injury. BBB breakdown occurs immediately due to direct stress-cell interactions, and further increase in BBB between injury and 4 hours can be attributed to oxidative stress. These events corroborate with the heightened activation of MMP-3 and MMP-9. Pretreatment with NOX inhibitor apocynin, BBB permeability is significantly reduced and the increase in MMPs levels were prevented. This study therefore indicates a strong relationship between NOX-mediated oxidative stress and blood brain barrier breakdown, which may lead to further investigation delineating relationships between other injury mechanisms, a prerequisite for better therapeutic interventions.
